# A prospective co-twin control analysis of internalizing and externalizing pathways for alcohol, nicotine, and cannabis use problems from adolescence through adulthood

**DOI:** 10.1017/S0033291726104802

**Published:** 2026-07-14

**Authors:** Katherine T. Foster, Daniel J. O. Roche, William G. Iacono, Matt McGue, Brian M. Hicks

**Affiliations:** 1 Psychology, University of Washington, Seattle, USA; 2 Global Health, University of Washington, Seattle, USA; 3 Psychiatry, University of Maryland Baltimore, USA; 4 Psychology, University of Minnesota Twin Cities, USA; 5 Psychology, University of Michigan, USA

**Keywords:** adolescence, adulthood, alcohol use, co-twin control, externalizing, internalizing

## Abstract

**Background:**

Alcohol, nicotine, and cannabis use problems have a poorer prognosis when they co-occur with internalizing distress (INT). One hypothesis to account for the association between INT and alcohol and substance use problems (SUP) is that people engage in heavy substance use to reduce aversive affective states characteristic of INT (e.g. affective reinforcement). Whether INT operates as a cause or a shared liability with SUP (e.g. shared genetic and environmental risk) over and above an alternative cause (e.g. externalizing problems, EXT) remains unclear.

**Methods:**

We tested INT as a developmental pathway for SUP by applying a prospective, co-twin control approach to the Minnesota Twin and Family Study sample (*n* = 1881 twin pairs). A multilevel modeling framework was used to estimate the between-twin pair effect and the within-twin pair effect of INT over and above EXT on separate SUP outcomes (i.e. alcohol, nicotine, and cannabis use problems) from adolescence (age 14) to young adulthood (age 29).

**Results:**

We failed to detect any non-familial causal effects of INT on SUPs. Instead, their associations were due to common genetic and shared environmental influences. In contrast, EXT had consistent non-familial causal influences on alcohol and cannabis use in adolescence and young adulthood.

**Conclusions:**

The link between INT and SUPs is primarily due to shared, familial influences while EXT likely serves as a unique determinant, with elevated EXT increasing selection into situations that pose SUP risk (e.g. peer environments that normalize and sustain heavy use) over and above common genetic influences.

## Introduction

Substance use problems (SUPs; e.g. alcohol, nicotine, and cannabis use problems) are commonly preceded by and co-occur with psychopathology symptoms on both the internalizing and externalizing spectra. Co-occurring psychopathology and SUPs involve greater impairment and poorer prognosis than SUPs without comorbid disorders (Bobo et al., 1998; Brown et al., 1995; Cornelius et al., 1995; Curran et al., 2000; Donohue et al., 1996; Driessen et al., 2001; Greenfield et al., 1998; Lejoyeux & Lehert, 2011; Meyer et al., 2004; Pettinati et al., 1997; Röggla & Uhl, 1995; Salloum et al., 1995). Consequently, both internalizing distress (i.e. INT: negative affect, inhibition, and symptoms of anxiety and depression) and externalizing behaviors (i.e. EXT: impulsivity, disinhibition, sensation-seeking, and antisocial behavior) have been characterized as pathways, mechanistic processes, or typologies through which specific risk increases SUPs onset and escalation in the period of adolescence through adulthood (Krueger, 1999; Krueger et al., 1998). Yet, there have been few *causal* tests of their association during the period of onset and escalation for SUPs.

Theories of SUP development posit that problem use is an equifinal outcome of cascading, interacting risks across multiple pathways (Cicchetti & Rogosch, 1996). One prominent hypothesis is that positive reinforcement (i.e. the euphoric, anxiolytic, or socially facilitating effects of substances) drives early substance use but repeated cycles of intoxication and withdrawal progressively shift toward negative reinforcement processes (Koob & Volkow, 2016; Wise & Koob, 2014). Across episodes of use and abstinence, negative reinforcement processes are expected to initially center on relief of withdrawal-driven hedonic dysregulation (Koob & Le Moal, 1997, 2008) but generalize to the mitigation of aversive affective states arising naturalistically outside the withdrawal context (Baker et al., 2004). Longitudinal evidence supports this transition: negative reinforcement becomes an increasingly dominant predictor of use as SUP develops, whereas positive reinforcement alone predicts use among those without symptoms (Cho et al., 2019).

Sustained affective distress characteristic of INT is expected to amplify this dynamic by entrenching substance use as a primary coping mechanism, eroding access to alternative reinforcers, and precluding more adventive coping strategies (Audrain-McGovern et al., 2011). Heavy sustained use in turn deepens withdrawal-driven regulation and further elevates affective burden, constituting a self-reinforcing cascade presumed to drive progression to SUP (Baker et al., 2004; Carpenter & Hasin, 1999; Koob & Le Moal, 1997; Koob & Le Moal, 2008; Koob & Volkow, 2016; Savage & Dick, 2023; Sinha, 2008; Stewart, 1996). Despite this theoretical coherence, longitudinal evidence for the INT-SUP link has been mixed: some studies report INT *increases* SUP risk (Boden & Fergusson, 2011; Crum et al., 2013; Kuo et al., 2006; Hussong et al., 2011), others report INT *reduces* SUP risk (Foster et al., 2018; Maggs et al., 2008; Reynolds et al., 2021; Steele et al., 1995), and still others report INT has a *weak or null effect* on SUP risk (Conegundes et al., 2023; Farmer et al., 2015, 2016; Miettunen et al., 2014). One source of inconsistency appears to be the developmental period: INT tends to protect against SUP in adolescence, as behavioral inhibition reduces peer substance exposure, but, by adulthood, INT becomes a risk for use frequency, quantity, and SUP symptoms (Colder et al., 2020; Rieselbach et al., 2023).

Alongside INT risk, a partially distinct EXT pathway additionally operates via early disinhibited, antisocial behavior amplified by trait sensation-seeking and heightened sensitivity to positive reinforcement, facilitating early substance exposure and selection into peer environments that normalize and sustain heavy use (Cloninger, 1987; Iacono et al., 1999; Krueger et al., 2002; Zuckerman & Kuhlman, 2000). EXT shows stronger, more consistent prospective effects on SUP than INT across substances and developmental periods (Conegundes et al., 2023; Farmer et al., 2015, 2016; Meque et al., 2019; Miettunen et al., 2014), though the relative contributions of INT and EXT vary meaningfully by developmental period and typology, substance type, and demographic characteristics (e.g. Bowen et al., 2022; Buu et al., 2012; Colder et al., 2020; Conway et al., 2018; Foster et al., 2014; Green et al., 2018; Hicks et al., 2010; Kendler et al., 2022; Rieselbach et al., 2023, 2024; Scalco et al., 2021; Zucker, 2006).

A critical interpretive challenge persists: observed INT-SUP and EXT-SUP associations may partly or wholly reflect a common liability shared among these constructs (e.g. shared genetic or familial confounds) rather than distinct causal effects of psychopathology on SUP risk (Kendler et al., 2003; Krueger et al., 2002). The INT-SUP association is further obscured by INT-EXT covariance, leaving unclear whether INT confers risk independently of EXT, whether their effects interact across development, or whether observed INT effects largely reflect shared liability with EXT (Colder et al., 2013; Foster et al., 2018; Hussong et al., 2011).

## Causal inference regarding psychopathology risk for SUPs

High rates of co-occurrence between psychopathology and SUPs have led to a ubiquity of *causal* assumptions regarding their associations. However, genetic or environmental risk shared by members of the same family may serve as a common cause of both. Familial risk for SUPs and psychopathology may be transmitted within a family from one generation to another through both biological (e.g. genetic sensitivity to alcohol’s effects) and social learning (e.g. learned coping behavior modeled by parents or peers) pathways (Easley & Epstein, 1991; Hussong & Chassin, 2004; Jarmas & Kazak, 1992; Morozova et al., 2014; Schuckit, 1994; Schuckit & Smith, 1997; Virtanen et al., 2021). While this is often hypothesized, no stringent causal tests across development, psychopathology constructs, and substances have been conducted to date to confirm that the psychopathology risk for SUPs is causal rather than attributable to *shared dynamics or processes* (e.g. shared genetic or environmental effects) that vary across people (i.e. within specific families) and time (i.e. at specific developmental periods).

A co-relative framework (Kendler, 2017; Kendler et al., 2016) implemented with domain-specific, developmentally-sensitive measures of psychopathology, and SUP offers specific advantages for testing causal effects across development by accounting for unmeasured variance shared within families. It permits direct testing of whether individual differences in risk exposure (e.g. INT) within a family predict SUP outcomes after being adjusted for the average level of that risk common to the family (i.e. shared genetic and environmental variation) at each developmental period. A longitudinal design strengthens this approach by establishing temporal ordering: earlier psychopathology risk predicts later SUP after adjusting for family-level liability, clarifying the direction of association at key developmental milestones for each substance.

**Figure 1.**
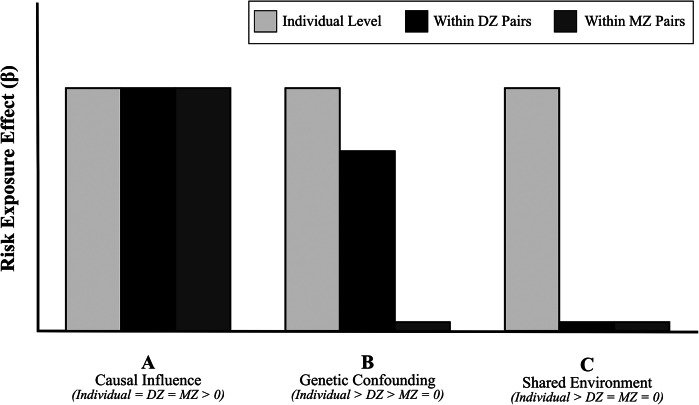
Illustration of interpretations for co-twin control model effects. *Note*: Co-twin control models involve comparison of individual-level effect parameters decomposed into separate parameters for a between-twin pair effect (i.e. the average level of the risk exposure within the family) and a within-twin pair effect (i.e. the unique risk exposure level each twin has relative to the family average). The between-twin pair effect permits covariate adjustment for the within-twin pair effect estimate, resulting in a within-twin pair effect that quantifies the variance in the outcome that is attributable to the unique influence of the risk on the outcome after adjusting for the degree of shared risk exposure within the family. Within-twin pair parameter estimates are then compared in twins with differing degrees of shared genetics (i.e. MZ and DZ twins that share roughly 100% and 50% of their genetics, respectively) to determine the source driving the risk exposure effect (e.g. the level of unique risk exposure itself, shared genetics, or shared environment). If the degree of risk exposure has a strong link with the outcome in twins with different degrees of shared genetics (i.e. the effect is strong and similar in MZ and DZ twins), the risk likely plays a causal role in the outcome as it is highly determinant of the outcome irrespective of the different contributions of genetic variation between twin types. Scenario A depicts a hypothetical causal influence outcome, wherein the DZ and MZ effects are both greater than zero and similar in magnitude. Alternatively, if the degree of extra risk exposure one twin experiences relative to their family average has a stronger link as a function of the degree of shared genetics (e.g. the effect is greater in DZ than MZ twins), the influence of the risk is confounded by genetics. Scenario B depicts a hypothetical genetic confounding outcome, wherein the influence of the risk exposure is attenuated after accounting for genetic similarity. Finally, if the between-twin pair risk exposure parameter (i.e. the average level of risk in the family) has a strong link with the outcome in the absence of strong within-twin pair effects for either twin type (Scenario C), the variance attributable to the risk variation in the outcome is likely due to the environment shared among families rather than the risk exposure itself or shared genetics.

Extending the co-relative framework to twin families (see Figure 1 for design assumptions) permits additional parsing of genetic or environmental contributions co-occurring factors (Irons, et al., 2012; Vitaro et al., 2009). Monozygotic (MZ) twins share all genetic material essentially, while dizygotic (DZ) twins share on average 50%, but twins of both types share their rearing environment when raised together. Differences within MZ pairs are therefore attributable to non-shared environmental influences or individual-specific risk exposures, whereas differences within DZ twins reflect both non-shared environment and genetic influences. If, for both MZ and DZ twins, the twin with higher psychopathology risk is also more likely to develop SUP, shared genetic and familial factors cannot explain the association – instead, non-shared processes such as selecting into higher-risk peer contexts or developing less effective coping strategies likely operate with causal influence, independently of the background liability both twins share. When applied prospectively (e.g. earlier INT predicts later SUP), the co-twin design identifies developmental periods when psychopathology risk is especially potent over and above shared familial liability.

## The present study

To address this aim, we applied a longitudinal, co-twin control design to isolate the prospective effects of psychopathology risk – separated across INT and EXT models after controlling for individual levels of the other – on alcohol, nicotine, and cannabis use problems after accounting for genetic and shared environmental effects. Using a community sample of families with twin offspring, the genetic similarities and differences between twins reared together were used to define our longitudinal co-twin control test across childhood (i.e. early initiation of substance use), adolescence (i.e. escalation of substance use), and young adulthood (i.e. period of peak prevalence of SUPs).

## Methods

### Sample

The Minnesota Twin and Family Study (MTFS) is an ongoing prospective community-based study designed to examine the etiology of alcohol and substance use disorders in twin families (Iacono & McGue, 2002; Iacono et al., 1999). Approximately 4057 parents (1965 fathers, 2092 mothers) and 3762 offspring (52% female; 1197 MZ and 684 DZ pairs) were initially recruited from the Minnesota Public Birth Records between 1972–1984 and 1988–1994. Participating families were almost exclusively of European–American ancestry and differed minimally from non-participating families in parental education, occupational status, or mental health. Following a cohort-accelerated design, two age cohorts were enrolled at different ages (i.e., ages 11 or 17, Iacono et al., 1999, Johnson et al., 2002) with follow-ups at 3–5-year intervals. Zygosity was assessed by parent-report on a standard zygosity questionnaire, staff evaluations of physical similarity, and confirmed by genome-wide genotyping (Iacono et al., 1999; McGue et al., 2013).

The present study utilized longitudinal data spanning 15 years of development with high retention rates (Johnson et al., 2002; Wilson et al., 2019), including ages 14 (*n* = 1,409), 17 (*n* = 2,557), 20 (*n* = 2,450), 24 (*n* = 2,499), and 29 (*n* = 2,500). Baseline assessments indexed lifetime and recent (e.g. past year) instances of use behavior and psychopathology and follow-ups thereafter captured time since last assessment and the past year. Thorough age-appropriate, psychometrically sound assessments (extensively described in Iacono et al., 1999) were scheduled to coincide with major developmental transitions in the lives of the adolescent and adult participants, including alcohol and substance use initiation (age 14), alcohol and substance use escalation (age 17), peak of problematic use (age 20), decline to moderate use (age 24), and stabilized patterns of desistence or persistence (age 29).

### Measures

#### Alcohol, nicotine, and cannabis use problems

Substance use was assessed using the Substance Abuse Module (SAM) of the Composite International Diagnostic Interview (Robins et al., 1988), supplemented with questions regarding use to assess symptoms of alcohol, nicotine, and cannabis use disorder as well as measures of quantity (i.e. number of intoxications, maximum number of alcohol drinks consumed in 24 hours, number of cigarettes per day, and number of cannabis uses per day) and frequency (i.e. number of days of nicotine and cannabis use) of use in the past year. Latent factors were developed for each substance at each age via confirmatory factor analysis (CFA) using a standard structure for SUPs: at least one indicator for use behavior (e.g. quantity or frequency) and one indicator for pathology (e.g. clinical symptoms of use disorder). Configuration was allowed to slightly vary by substance (e.g. use of frequency instead of quantity) to ensure sensitivity to developmental change in markers of risk (e.g. frequency of drinking alcohol marks high risk earlier in development than later). For the alcohol use problem (AUP) factor, the latent variable indicators included alcohol use disorder symptoms, number of intoxications, and maximum number of drinks in 24 hours. For the nicotine use problem (NUP), the latent variable indicators included nicotine use disorder symptoms, cigarettes per day, and number of days of cigarette use. For the cannabis use problem (CUP) outcome, the latent variable indicators included cannabis use disorder symptoms and number of days of cannabis use.

#### Internalizing and externalizing problems

Psychopathology was assessed using the Structured Clinical Interview for DSM (SCID) and self-report on dimensional measures of domain-relevant problems. Integrating this interview and self-report data, levels of INT and EXT were estimated via CFA models for each spectrum at each age. Indicators of the INT factor a symptom counts for major depressive disorder from the SCID for DSM-III-R along with self-reported stress reactivity and alienation factor scores of the Multidimensional Personality Questionnaire (Patrick et al., 2002; Tellegen & Waller, 2008) which has been used extensively with adolescents (Blonigen et al., 2008; Durbin et al., 2016; Johnson et al., 2007). Indicators of the EXT factor included the constraint factor score of the MPQ and an age-specific symptom indicators (i.e. symptoms of conduct disorder at age 14 and symptoms of the adult criteria for antisocial personality disorder from the ASPD module of the SCID for DSM-IV at all other ages).

### Statistical analysis

Successive developmental models were fit using data from age 14 through age 29 to test whether there is a non-familial causal effect of psychopathology risk factors (INT and EXT) on SUP outcomes (i.e. AUP, CUP, and NUP) at each SUP inflection point from adolescence through young adulthood (i.e. childhood to early adolescence, early adolescence to later adolescence, later adolescence to young adulthood). CFAs for all constructs exhibited excellent fit regarding established cut-offs (i.e. Brown, 2014: root mean square error of approximation, RMSEA < 0.05; standardized root mean square residual, SRMR < 0.05; comparative fit index, CFI > 0.95; and non-normed fit index, NNFI > 0.95). Latent factor scores were derived from each CFA and submitted to separate, lagged multilevel models. Each model had the same general longitudinal structure with psychopathology risk exposure estimated at the younger assessment (e.g. age 20) and the SUP outcome at the next assessment (e.g. age 24) to capture time-specific effects in the developmental SUP trajectory rather than estimating a single, linear effect across the entire period that would occlude transitions.

#### Regression framework for co-twin control analyses

Using a co-twin control multilevel modeling framework (Begg & Parides, 2003), the average effect of the psychopathology risk factor on the SUP outcome across all individuals was compared to estimates of the unique effects of the risk factor for individuals over and above the average amount of risk common to both twins (i.e. the family-level effect). For each model, SUP at the later age was regressed on earlier psychopathology risk (i.e. the individual-level effect) using the following equation:

SUPij=
β
0+
β
IND∗INTRISKij+
ε
ij

This model estimates SUP for the individual **j** in the **i**th twin pair by combining three estimated effects: the average level of the SUP outcome when the INT risk is zero (**β**_**0**_), the effect of individual variation in the INT risk (**β**_**IND**_), and measurement error (**ε**_**ij**_).

#### Co-twin models

The resulting individual level psychopathology risk effect (e.g. **β**_**1**_ **INT RISK**_**ind**_ from the model above) was then decomposed in subsequent models to separate estimates of the effect of *unique* and *shared* levels of the risk within each twin family. For these models, the regression of SUP on earlier psychopathology risk was decomposed into a within-pair INT risk effect (**β**_**W**_) and a between-pair INT risk effect (**β**_**B**_) as follows:

$$\begin{array}{l} {SUP}_{ij}={\text{β}}_{0}+{\text{β}}_{W}*\left(INT\;{RISK}_{ij}\text{ - }{\bar{INT\;RISK}}_{i}\right)\\ \;+{\text{β}}_{B}*\left({\bar{INT\;RISK}}_{i}\right)+{\text{ɛ}}_{ij} \end{array}$$

The **β**_**B**_ effect is the estimate for the average amount of the INT risk shared by twins in the same family (i.e. the risk value for Twin A added to risk value for Twin B divided by two). The **β**_**W**_ effect (the primary focus of this study) is the amount of INT risk unique to the twin (i.e. risk value for Twin A – twin average for risk). Consequently, this parameter models the difference in outcome between the members of a twin pair who vary in the degree of INT risk they report at the earlier age after adjusting for the INT risk (and its unmeasured corresponding features) that are common to both twins in the same family (**β**_**B**_).

The final model also included an alternative psychopathology risk covariate (e.g. following the example above, **EXT RISK**_**ij,**_ to account for the effect of individual variation in EXT), a **Zygosity** status parameter to control for potential differences between twin types (MZ versus DZ) in the outcome, and an interaction term between **Zygosity x INT RISK**_**w**_ to estimate differential effects of unique INT risk across the different twin types. The resulting final model following the example above was:

SUPij=
β0+
β
ZYG+βW∗(INTRISKij - INTRISK¯i)+βB∗(INTRISK¯i)+
β
ZYG∗W+
β
ZYG∗B+
β
IND∗EXTRISKij+
ε
ij

Alternative models were also fit to test to decompose the effect of the alternate risk – EXT – on each of the SUP outcomes after adjusting for concurrent level of INT. Consequently, the resulting final alternative models were structured as the example below, with separate models for each SUP outcome:

SUPij=
β
0+
β
ZYG+βW∗(EXTRISKij - EXTRISK¯i)+βB∗(EXTRISK¯i)+
β
ZYG∗W+
β
ZYG∗B+
β
IND∗INTRISKij+
ε
ij

#### Inferences from the co-twin model

The pattern of results across MZ and DZ can be used to discern the predominant influence on the association between the INT risk and each of the SUP outcomes (i.e. separate models for each SUP outcome) over and above the individual level of EXT risk **β**_**IND**_ (i.e. scenarios are presented in Figure 1). For example, a *causal effect* of INT risk on the AUP outcome would be indicated by a pattern of results including a significant **INT**_**w**_ **risk** effect for both MZ and DZ pairs (Figure 1A). That is, if the INT risk has the same effect irrespective of the amount of shared genes and environment (i.e. similar effect of across twin pair types), INT can be said to have a direct, causal influence on the AUP outcome. Alternatively, a non-significant **INT**_**w**_ **risk** effect in MZ pairs and a significant or larger **INT**_**w**_ **risk** effect in DZ pairs would indicate *genetic confounding* (Figure 1B). That is, controlling for shared genetics would reduce the magnitude of the effect (lower in MZ twins because they share more genes than DZ twins). A non-significant **INT**_**w**_ **risk** effect for both MZ or DZ pairs would provide evidence for a shared environmental influence (Fig. 1C). That is, a shared environment would account for the AUP-INT link if no effect is present in either the DZ or MZ twins (who share the same environment), which would eliminate the INT effect on AUP.

## Results

Results of multilevel co-twin control model estimation of variation in AUP, NUP, and CUP outcomes attributable to lagged effects of INT and EXT from age 14 to age 29 are presented in Table 1. As the focal inferences of this work are drawn across sets of effects in the same model (i.e. the decomposition of the individual effect into within pair effects for each twin type) and between models within each substance (i.e. patterns of effects across development) labeled interpretations based on the co-twin control framework (i.e. attributable to causal link, genetic confounding, or shared environmental factors) are also included. Model results are discussed below, clustered by SUP outcome and psychopathology risk.

**Table 1.** Prospective effects of psychopathology risk on later addictive behavior outcomes Table 1. long description.

	Early adolescent model		Older adolescent model		Young adult model		Adult model	
	(Age 14 - > 17)		(Age 17 - > 20)		(Age 20 - > 24)		(Age 24 - > Age 29)	
	*β*	SE	*β*	SE	*β*	SE	*β*	SE
**Predicting alcohol problems**								
*Zygosity*	−0.10	0.05	−0.10	0.08	0.00	0.07	−0.07	0.05
*Internalizing*								
*INT_b_*	0.04	0.02	0.01	0.03	0.09***	0.03	−0.01	0.02
*INT_w_ – DZ*	−0.09	0.07	0.00	0.06	0.14*	0.06	0.03	0.05
*INT_w_ – MZ*	−0.05	0.04	−0.14	0.10	−0.04	0.07	−0.04	0.06
**Interpretation**	***No effect***		***No effect***		**Genetic confounding**		***No effect***	
*Externalizing*								
*EXT_b_*	0.26***	0.03	0.29***	0.04	0.28***	0.03	0.43***	0.02
*EXT_w_ – DZ*	0.23**	0.07	0.19*	0.09	0.22**	0.07	0.48***	0.06
*EXT_w_ – MZ*	0.03	0.05	0.15*	0.07	0.14*	0.07	0.33***	0.06
**Interpretation**	**Genetic confounding**		**Causal +** **Partial genetic confounding**		**Causal +** **Partial genetic confounding**		**Causal + Partial genetic confounding**	
**Predicting nicotine problems**								
*Zygosity*	−0.13	0.07	−0.15	0.08	−0.19	0.08	0.01	0.09
*Internalizing*								
*INT_b_*	0.04	0.03	0.09**	0.04	0.13***	0.03	0.06	0.04
*INT_w_ – DZ*	0.00	0.06	−0.23*	0.11	0.12	0.09	0.17	0.10
*INT_w_ – MZ*	−0.01	0.04	0.06	0.06	0.05	0.06	−0.01	0.07
**Interpretation**	***No effect***		**Genetic confounding**		**Environmental**		***No effect***	
								
*Externalizing*								
*EXT_b_*	0.32***	0.03	0.30***	0.04	0.29***	0.04	0.40***	0.04
*EXT_w_ – DZ*	0.23***	0.06	0.31**	0.09	0.34***	0.09	0.39***	0.10
*EXT_w_ – MZ*	0.08	0.05	0.11	0.07	0.13*	0.06	−0.09	0.08
**Interpretation**	**Genetic confounding**		**Genetic confounding**		**Causal +** **Partial genetic confounding**		**Genetic confounding**	
								
**Predicting cannabis problems**								
*Zygosity*	−0.11	0.07	−0.08	0.06	−0.08	0.07	−0.14*	0.06
*Internalizing*								
*INT_b_*	0.02	0.03	−0.03	0.03	0.00	0.03	0.02	0.03
*INT_w_ – DZ*	−0.04	0.08	−0.16	0.09	0.10	0.10	−0.11	0.07
*INT_w_ – MZ*	−0.10	0.05	−0.02	0.04	−0.03	0.04	0.02	0.04
**Interpretation**	***No effect***		***No effect***		***No effect***		***No effect***	
								
*Externalizing*								
*EXT_b_*	0.25***	0.03	0.20***	0.03	0.25***	0.04	0.25***	0.03
*EXT_w_ – DZ*	0.09	0.09	0.09	0.08	0.20*	0.10	0.18*	0.07
*EXT_w_ – MZ*	0.15*	0.06	0.1	0.06	0.18***	0.05	0.16**	0.05
**Interpretation**	**Causal +** **Partial genetic confounding**		**Environmental**		**Causal +** **Partial genetic confounding**		**Causal + Partial genetic confounding**	

*Note*: * = *p* < 0.05, ** = *p* < 0.01, *** = *p* < 0.001; INT_b_, between-pair effect, INT_w_, within-pair effect.

### AUP outcome

#### INT risk for AUP

After adjusting for the prospective effects of EXT (i.e. β_IND_* EXT RISK_ij_), the prospective individual-level effect of INT was only significant for AUP at age 24 (Table 1) and the decomposition of these effects into within-pair DZ and MZ effects suggests genetic confounding (i.e. INT*_b_* > DZ*_w_* > MZ*_w_*). Apart from this small influence of INT at age 20 on AUP at age 24, the prospective effects of INT on AUP were consistently non-significant and around zero from age 14 through age 29, suggesting that INT is not a stable antecedent for AUP after adjusting for the effects of EXT, which were consistent and notable across the entire period (i.e. significant EXT covariate in each model).

#### EXT risk for AUP

After adjusting for the individual prospective effects of INT (i.e. β_IND_*INT RISK_ij_), the prospective individual-level effects of EXT on AUP were significant and moderate in size (*β* range 0.26–0.43) in all lagged models from age 14 to age 29 (Table 1). The individual prospective effect of EXT on AUP was stable until age 24, when it escalated through age 29, suggesting that, with age, EXT is a stronger predictor of AUP. Prospective EXT effects were consistently attributable to a causal effect with genetic confounding (EXT*_b_* > DZ*_w_* > MZ*_w_*) at each outcome age, with effects peaking and converging most (EXT*_b_* = DZ*_w_* = MZ*_w_*) for AUP at age 29, suggesting EXT may play an increasingly causal role (i.e. declines in partial genetic confounding) in AUP.

### NUP outcome

#### INT risk for NUP

After adjusting for the individual prospective effects of EXT (i.e. β_IND_*EXT RISK_ij_), the prospective individual-level effect of INT on NUP was small but significant at ages 20 and 24 (Table 1). Decomposition of these effects across twin types suggests genetic confounding for age 20 (i.e. the magnitude of the within-DZ effect exceeds the within-MZ effect) and familial confounding (i.e. either genetic or shared environmental confounding) by age 24 (i.e. between-pair effect is significant, but the within-pair effect is not significant in either twin type). Apart from this confounding, the prospective effects of INT on NUP were otherwise consistently near zero from age 14 through age 29 after adjusting for the prospective effects of individual-level EXT.

#### EXT risk for NUP

After adjusting for the individual prospective effects of INT (i.e. β_IND_* INT RISK_ij_), the prospective individual-level effects of EXT on NUP were significant and consistently moderate in size (*β* range 0.29–0.40) from age 14 to age 29 (Table 1). Decomposition of these effects detected only a small, within-pair effect at age 24, suggesting some causal role of EXT at this age. However, the prospective effects of EXT on NUP after adjusting for INT appear to be largely due to genetic confounding from age 14 through and 29.

### CUP outcome

#### INT risk for CUP

After adjusting for the individual prospective effects of EXT (i.e. β_IND_*INT RISK_ij_) on CUP, the prospective individual-level effect of INT on CUP was non-significant and near zero across all models (Table 1).

#### EXT risk for CUP

After adjusting for the individual prospective effects of INT (i.e. β_IND_*EXT RISK_ij_), the prospective individual-level effects of EXT on CUP were consistently significant and moderate in size (*β* range 0.20–0.25) from age 14 to age 29 (Table 1). Importantly, the prospective effects of EXT on CUP appear to be attributable to causal influences with some genetic confounding at ages 17, 24, and 29 and genetic confounding at age 20.

## Discussion

Using longitudinal, community sample data spanning adolescence through adulthood, we applied a co-twin control design to identify sources of variation in AUP, NUP, and CUP attributable to earlier INT and EXT symptoms. Chief innovations of this work were the use of a longitudinal, behavior genetic framework, segmentation of models by key inflection points in the development of addiction, and effects comparison across and accounting for psychopathology spectra. While other twin studies estimate heritability in SUP risk and growth rates (e.g. King et al., 2004; Malone et al., 2021; Zellers et al., 2020), the present study uniquely used one twin as a control comparison for the other, subtracting out shared genetic and environmental confounds by design, to predict the degree to which psychopathology (INT versus EXT) operates as a prospective cause of SUP vulnerability across key periods of developmental when use patterns typically shift.

Despite previously documented associations between both INT and EXT and each SUP in the broader literature, the present test of a causal relationship was supported between SUP and EXT but not INT. EXT remained a robust predictor of SUP across ages and substance types but the effect of INT on each SUP was consistently diminished, eliminated, and even reversed (e.g. suppression effects; Foster et al., 2018) following inclusion of the alternative EXT factor. In the few cases where INT exerted a significant influence on the SUP (e.g. INT at age 20 predicts AUP at age 24), risk within twin pairs signaled shared genetic effects, rather than causal determinants. Overall, results across developmental models provide strong evidence that EXT poses developmentally-consistent, causal risk for each SUP over and above the parallel prospective influence of INT and that common comorbidity between INT and SUPs is stems from shared genetic (i.e. non-causal) influences.

The observation of a general genetic liability for SUPs spanning multiple developmental periods (Dick, 2022; Hicks et al., 2004; Pagan et al., 2006; Verhulst et al., 2015) suggest SUP outcomes may stem from the developmentally cascading causal operations of EXT but not INT processes. The null INT effect is consonant with recent findings from rigorous daily-life studies testing the link between negative emotionality and alcohol use (Dora et al., 2023). Models positing that static, trait-level affective experiences drive SUP via INT pathways may not operate causally across people in the prevailing theories assume. Historically, the INT-SUP relationship has been estimated by averaging effects across individuals, which may obscure meaningful heterogeneity in pathways leading to SUP. High rates of INT-SUP co-occurrence may therefore reflect shared antecedents – genetic liability, non-shared environmental influences, or EXT – rather than a direct causal relationship, with some SUP cases involving no meaningful INT elevation at all as prior typological and developmental trajectories work on internalizing pathways has suggested.

The present study has several limitations. The sample was comprised of individuals of European-American descent during the transition from adolescence through young adulthood, potentially limiting the generalizability of results to diverse groups or developmental periods. While the present use of a substance-specific, developmentally-sensitive, dimensional framework strengthens the precision and innovation of this work, models were optimized to substance-specific inferences, not draw equivalence *between* substance use types – any single numeric value is not expected to index the equivalent clinical severity across SUPs, and results constitute separate, independent tests of co-twin control effects for each SUP. A related assumption is that results generalize uniformly across the full continuum of severity; however, the present analytic approach estimates a single linear effect of psychopathology risk that may not capture differential effects across severity levels – for instance, INT may predict AUP more strongly among those with severe problems than among light drinkers (Cho et al., 2019), and families with greater genetic aggregation of both AUP and INT risk may show relatively more genetic confounding than those with less shared overlap. Additionally, models tested prospective reinforcement across years rather than the short-term reinforcement dynamics operating as a function of the pharmacological effects of the substance. Consequently, while lagged models permitted estimation of INT and EXT earlier in development as predictors of later SUP, they are not equipped to test relief as a short-term consequence of drug cues or specific occasions of use. Finally, while the co-twin control design permits a strong test of causal inference, results should be interpreted with caution until replicated.

Future work would benefit from deeper consideration that INT and EXT co-occurrence with SUP may operate causally only under certain conditions: in some individuals but not others, at granular moment-to-moment timescales rather than across persisting affective states, in some developmental periods but not others, and for some operationalizations of psychopathology but not others (e.g. transdiagnostic spectra versus granular DSM symptoms or profiles). Gueguen et al. (2021) specifically encourage computational approaches for capturing reinforcement cycles and states dynamically within-person across time, which would address several of these conditions simultaneously. Future work could also extend the co-twin control framework to test bidirectional effects, examining (1) whether substance exposure at key developmental periods causally influences subsequent psychopathology and (2) patterns of co-use and polydrug symptom status, which may show differential associations with psychopathology risk (e.g. EXT may have stronger causal links with indiscriminate use of available substances).

Despite extensive evidence linking INT, EXT, and SUPs, this study is the first to chart whether these associations reflect familial confounding of the causal processes that models of addictive behavior most commonly present. While some work suggests that INT may be protective earlier in development, the present results suggest that this effect may be weak and is likely not playing a causal role in mitigating SUP development. The detection of EXT but not INT as a causal influence at multiple points in the continuum of developing alcohol, nicotine, and cannabis use problems suggests that early identification and treatment of EXT may be a particularly high-yield target for reducing SUP onset, severity, and persistence across the transition to adulthood. Critically, the EXT reduction may be effective irrespective of the shared familial liability for psychopathology.

## Long descriptions

### Table 1. Long description

The table is divided into three primary sections predicting alcohol, nicotine, and cannabis problems. Each section includes beta values and S E for four models: Early Adolescent (Age 14 to 17), Older Adolescent (Age 17 to 20), Young Adult (Age 20 to 24), and Adult (Age 24 to 29).

* Predicting alcohol problems: Internalizing factors show mostly no effect, except for genetic confounding in the Young Adult model. Externalizing factors show genetic confounding in early adolescence, transitioning to causal plus partial genetic confounding from older adolescence through adulthood, with beta values increasing from 0.26 to 0.43.

* Predicting nicotine problems: Internalizing factors show no effect or genetic confounding, except for an environmental effect in the Young Adult model. Externalizing factors show genetic confounding in most stages, with causal plus partial genetic confounding appearing in the Young Adult model (beta 0.29).

* Predicting cannabis problems: Internalizing factors show no effect across all age models. Externalizing factors show causal plus partial genetic confounding in early adolescence (beta 0.25), an environmental effect in older adolescence (beta 0.20), and return to causal plus partial genetic confounding in young adulthood and adulthood (beta 0.25).

Statistical significance is noted where p is less than 0.05, 0.01, or 0.001. Variables include Zygosity, I N T sub b (between-pair effect), and I N T sub w (within-pair effect) for both D Z (dizygotic) and M Z (monozygotic) twins.

Navigate back to Table 1..
